# TST36 stapling for rectocele and hemorrhoidal prolapse – early results of the prospective German multicenter study

**DOI:** 10.3205/000241

**Published:** 2016-12-19

**Authors:** Sven Petersen, Daniel Sterzing, Andreas Ommer, Assen Mladenov, Zrino Nakic, Faramaz Pakravan, Katja Wolff, Eric P. M. Lorenz, Ruediger L. Prosst, Marco Sailer, Roland Scherer

**Affiliations:** 1 Department of General, Visceral and Vascular Surgery, Hospital Asklepios Altona, Hamburg, Germany; 2 Center of Colorectal and Pelvic Floor Surgery, Hospital Waldfriede, Berlin, Germany; 3 Center of Coloproctology Essen, Germany; 4 Center of Proctology Gendarmenmarkt, Berlin, Germany; 5 Center of Coloproctology, Duesseldorf, Germany; 6 Department of Visceral and Vascular Surgery, St. Hedwig Hospital Berlin, Germany; 7 Proctological Institute Stuttgart, Germany; 8 Department of Surgery, Bethesda General Hospital Bergedorf, Hamburg, Germany

**Keywords:** stapled surgery, hemorrhoids, rectocele, obstructed defecation, stapled hemorrhoidectomy, prolapse

## Abstract

**Introduction:** The aim of the study was to evaluate the safety and feasibility of stapled transanal procedures performed by a 36 mm stapling device, the so-called TST36 stapler.

**Methods:** From September 2013 to June 2014 a prospective observational study was carried out by 8 proctology centers in Germany. The Cleveland Clinic Incontinence Score (CCIS) for incontinence and the Altomare ODS score were determined preoperatively. Follow-up examinations were performed after 14 days, one month and 6 months, at this time both scores were reevaluated.

**Results:** 110 consecutive patients (71 women, 39 men) with a mean age of 59.7 years (±13.8 years) were included in the study. The eight participating institutes entered 3 to 31 patients each into the study. The indication for surgery was an advanced hemorrhoidal disease in 55 patients and ODS with rectal intussusception or rectocele in 55 patients. Mechanical problems with stapler introduction occurred in 22 cases (20%) and a partial stapleline dehiscence in 4 cases (3.6%). Additional stitches for bleeding from stapleline were necessary in 86 patients (78.2%). Reintervention was necessary for bleeding 7 times (6.3%). Severe complications during follow-up were stapleline dehiscence in one case and recurrent hemorrhoidal prolapse in 5 cases (4.5%). Altomare ODS score and CCIS improved significantly after surgery.

**Conclusions:** Despite a notable complication rate during surgery and the postoperative period, the TST36 can be considered as an effective tool for low rectal stapling for anorectal prolapse causing hemorrhoids or obstructed defecation.

## Zusammenfassung

**Einführung:** Das Ziel der Studie war es, die Sicherheit und Durchführbarkeit der transanalen Klammernahtoperation mit einem neuen 36 mm Stapler, dem so genannten TST36-Stapler zu bewerten.

**Methoden:** Ab September 2013 bis Juni 2014 wurde eine prospektive Beobachtungsstudie von 8 Proktologie-Zentren in Deutschland durchgeführt. Der Cleveland Clinic Inkontinenz-Score (CCIS) für Inkontinenz und der Altomare-ODS-Score wurden präoperativ bestimmt. Follow-up-Untersuchungen wurden nach 14 Tagen, einem Monat und 6 Monaten durchgeführt. Zu diesen Zeitpunkten wurden beide Scores erneut evaluiert.

**Ergebnisse:** 110 Patienten (71 Frauen, 39 Männer) mit einem Durchschnittsalter von 59,7 Jahre (±13,8 Jahre) wurden in die Studie einbezogen. Die acht teilnehmenden Institute rekrutierten jeweils zwischen 3 bis 31 Patienten. Die Indikation zur Operation war ein fortgeschrittener Hämorrhoidalprolaps bei 55 Patienten und ODS mit rektale Invagination oder Rektozele bei 55 Patienten. Mechanische Probleme beim Einführen des Staplers gab es in 22 Fällen (20%) und eine partielle Klammernahtdehiszenz in 4 Fällen (3,6%). Zusätzliche Umstechungen bei Blutungen aus Klammernahtreihe waren bei 86 Patienten (78,2%) erforderlich. Wegen Blutungen waren insgesamt 7 Reinterventionen (6,3%) notwendig. Als schwere Komplikationen während des Follow-up wurden eine Klammernahtdehiszenz und rezidivierender Hämorrhoidalprolaps in 5 Fällen (4,5%) beobachtet. Altomare-ODS-Score und CCIS waren nach der Operation deutlich verbessert.

**Schlussfolgerungen:** Trotz einer offenkundigen peri- und postoperativen Komplikationsrate kann die TST36-Stapleroperation als wirksame Maßnahme zur Behandlung anorektaler Prolapsformen und einer obstruktiven Defäkationsstörung angesehen werden.

## Introduction

During the last two decades stapling procedures for anorectal prolapses and functional disorders of the lower rectum became an approved treatment option. Since stapled hemorrhoidopexy was introduced in 1998 it was proven to be a save procedure and early results were superior to those of conventional surgery. In 2002 the transanal stapled rectal resection (STARR) was introduced and the indication was enlarged to obstructed defecation syndrome (ODS) and rectocele [1], [2]. For this, the standard STARR technique uses two PP01 staplers (Ethicon Surgery, Johnson & Johnson, Norderstedt, Germany) [3], [4], [5]. 

**Figure 1:**
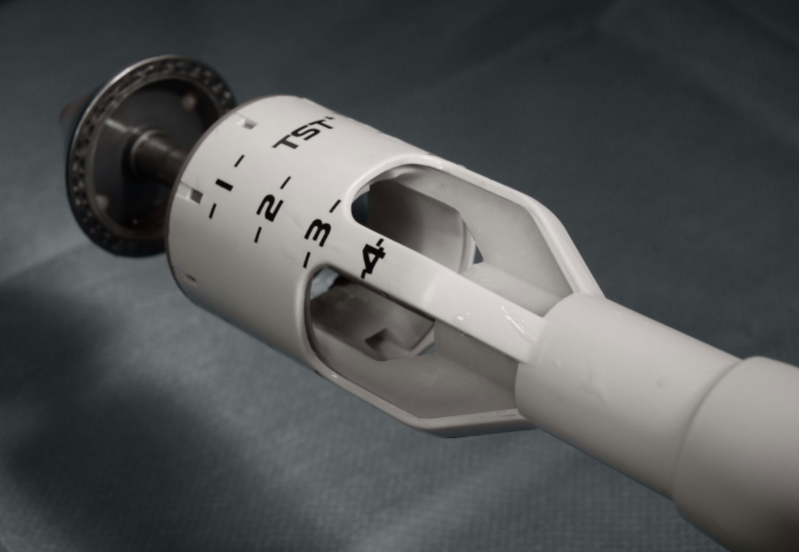
TST36 stapler with large size windows for optimal visualization during the procedure and large specimen housing, with a volume of up to 35 ml

Recently a new stapling device was introduced from China by Touchstone (Touchstone International Medical Science Co. Ltd., Suzhou, China). This device, called TST36 has two main features which set it apart from other stapling devices: very large size window, designed to provide optimal visualization during the procedure and this device also has the largest specimen housing, with a volume of up to 35 ml to allow for larger resections of tissue (Figure 1 (Fig. 1)). In contrast to the traditional STARR procedure with two staplers, the TST36 stapling procedure offers the opportunity to perform a low rectal wall resection with only one stapling device.

## Material and methods

In the period from September 2013 to June 2014 a prospective observational study was carried out by 8 proctology centers in Germany. All patients signed informed consent on study participation. An acknowledgement for this analysis was supplied by the local ethics committee (Ethik-Kommission der Ärztekammer Hamburg, Nummer WF-61/16). 

### Inclusion

Both, patients with large third degree hemorrhoids and/or an obstructed defecation syndrome (ODS) with internal rectal prolapse were included into the study. Being aware, that isolated forms of hemorrhoidal prolapse or obstructed defecation forms are uncommon, surgeon was requested to decide whether the primary problem is hemorrhoids or obstructed defecation.

### Exclusion

The study excluded patients with previous rectoanal operations and in particular patients who already had stapled procedures or who suffered from relevant comorbidities. 

Preoperatively, the results of a clinical examination and an endosonography of the sphincter apparatus were at hand. An anal manometry was only optional. The Cleveland Clinic Incontinence Score (CCIS) for incontinence and the Altomare ODS score was determined preoperatively [6]. Both scores were determined at follow-up one and six months after surgery. The pain score was recorded using numerical and visual analogue scale, VAS (0–10). 

Follow-up examinations were performed after 14 days, one month and 6 months. Hemostatic sutures for bleeding of the staple suture line, operation time, hospital-stay and perioperative complications were recorded. The gradual reduction in hemorrhoidal prolapse was evaluated by the surgeon’s suggestion.

### Data collection 

Disease history and clinical status were recorded from referral letter, surgical and anaesthesia admission chart. Histologic aspects were collected from the pathologic specimen report. Outcome including postoperative complications, partial or total wound dehiscence, and wound progress and discharge status were identified from clinical progress notes and cross-checked with the discharge letter. The complete study record of these patients was acquired within 6 months.

### Statistical methods

Statistical analysis was carried out using the Statistical Package for the Social Sciences^®^ for Windows^®^ Version 18.0 (SPSS Corp., Chicago, Ill., USA), Pearson chi-square-test compared the incidence of variables. Parameters, which were not suitable for the Pearson chi-square-test, were evaluated using the T-test; these variables were summarized as mean and standard deviation. Correlation was calculated using 2-tailed Pearson correlation-test. Variables with p-value less than 0.05 were considered to be significant.

### Surgical technique

The procedure was carried out either in general anesthesia or spinal anesthesia in lithotomy position. Single shot antibiosis was not given routinely. For stapling the TST36 was used. The TST36 stapler (Touchstone International Medical Science Co. Ltd., Suzhou, China) has a housing length of 6 cm with an internal volume of more than 35 cm^3^ and a blade diameter of 28 mm and a housing diameter of 36 mm. It carries 34 staples with a height of 4.2 mm in open condition and a closure range of 0.75–1.8 mm (Figure 1 (Fig. 1)). The rectal cuff could be drawn into the stapler either by a purse-string suture or by parachute sutures. Additional stitches for hemostasis were performed regularly at the stapled ring using monofilament resorbable sutures. All operations were performed by experienced proctologist who had previously received appropriate training. Surgeons in all participating centers were familiar with conventional STARR procedure with two PPH01 stapling devices. 

Postoperative routinely non-steroid analgetics were administered during hospital stay. If analgetics escalation to morphine-derivates was necessary, this was recorded as “additional analgetics”.

Patients left the hospital as soon as they felt comfortable. In no case the procedure was undertaken in an out-patient setting.

## Results

From September 1^st^ 2013 to June 30^t^h 2014, 110 consecutive patients (71 women, 39 men) with a mean age of 59.7 years (±13.8 years) were included in the study. The eight participating institutes entered 3 to 31 patients each into the study. Using chi-square test, an inhomogeneity according to patient number and indication was observed within the recruiting hospitals (p>0.01) (Table 1 (Tab. 1)). 

The indication for surgery was an advanced hemorrhoidal disease in 55 patients and ODS with rectal intussusception or rectocele in 55 patients. Only 14 patients had previously undergone gynecologic surgery (9 hysterectomies, 4 a tension-free vaginal tapes (TVT) and one colpoperineoplasty). 

The average duration of the procedure was 30.2 (±9.9 min) with a mean hospital stay of 3.5 days (±1.3 days). The mean specimen weight was 14.3 g (±4.6 g) with a mean length of the specimen of 4.3 cm (±1.1 cm). 

After firing the stapler, for minor bleedings in addition a mean of 2 hemostatic stitches into the stapleline were necessary in 86 patients (78.2%). In 4 cases an intraoperative partial dehiscence of the stapler suture occurred, which had to be sutured. 3 intraoperative hematomas occurred; all affected patients with history anticoagulation drugs. According to the recruiting institute, no correlation was found between complication rate and the recruiting hospital in the chi-square test (p=0.66).

Considering the postoperative complications during the first two weeks postoperatively, there were 4 postoperative hemorrhages; in two of them a surgical revision was necessary. One staple suture dehiscence was treated by endoluminal vacuum therapy. 4 weeks after the operation, one hemorrhage occurred, which had to be amended surgically. The overview on complications is shown in Table 2 (Tab. 2).

Overall, 7 patients were revised surgically for postoperative hemorrhages (6.3%) and 1 hemorrhoidectomy for hemorrhoidal thrombosis took place. In addition, 5 patients underwent hemorrhoidectomy for residual prolapse and/or early recurrence (4.5%). There was also no correlation between the recruiting hospital and the postoperative complication rate (p=0.20).

Considering fecal urgency, in the four week control, 22 patients stated urge symptoms with reduced warning time for defecation (21%), after 6 months these symptoms were complained by only 5 patients (4.8%). Fecal incontinence did not occur. 

The total mean preoperative VAS value was 0.7 (±0.9) with values of 3.1 (±1.7) and 2.7 (±1.7) at 6 h and 24 h. 24 hours after surgery, 39 patients (38%) needed additional analgetics during the hospital stay.

In 2 weeks, the average VAS score was 1.9. 38 patients (37%) were still taking analgetics. In a month, the average VAS score was 0.8. 9 patients (8%) were still taking analgetics. Six months after surgery, no patient complained of pain at the surgical area. 

**Figure 2:**
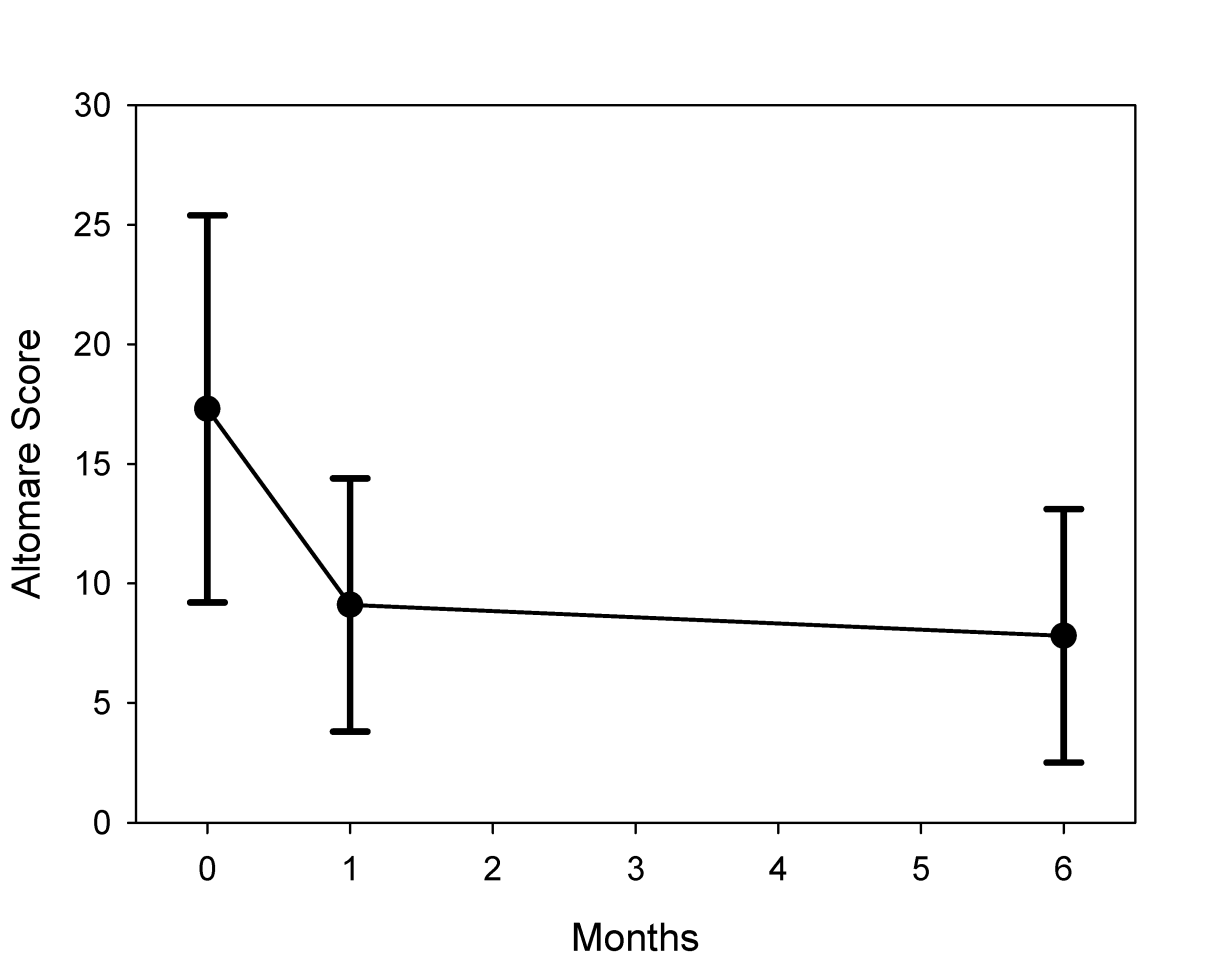
Altomare score for outlet obstruction preoperative and at 1 month and 6 month follow-up

**Figure 3:**
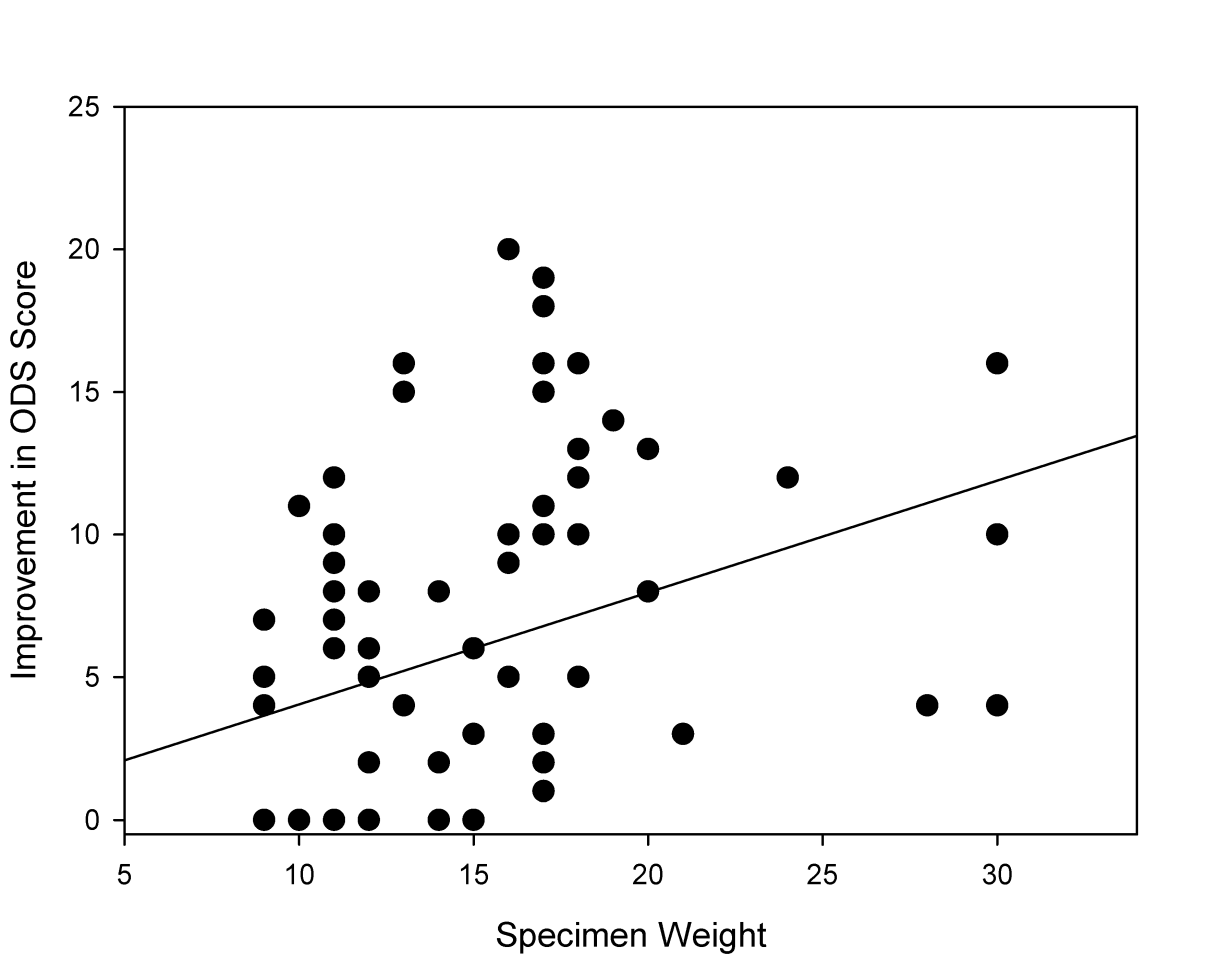
Correlation of improvement in the Altomare score and specimen weight

The Altomare constipation score performed in all patients decreased from 12.2 (±9.2) preoperatively to 4.3 (±5.4) after one month and 5.3 (±3.7) after 6 months. The impact of surgery was even more pronounced in the subgroup analysis for obstructed patients only. In these patients, the ODS score decreased from 17.3 before surgery to 9.1 after 1 month (p<0.01) (Figure 2 (Fig. 2)). A close correlation between the amount of resected rectal wall tissue and improvement of ODS was found (p=0.01, R=0.3) (Figure 3 (Fig. 3)).

**Figure 4:**
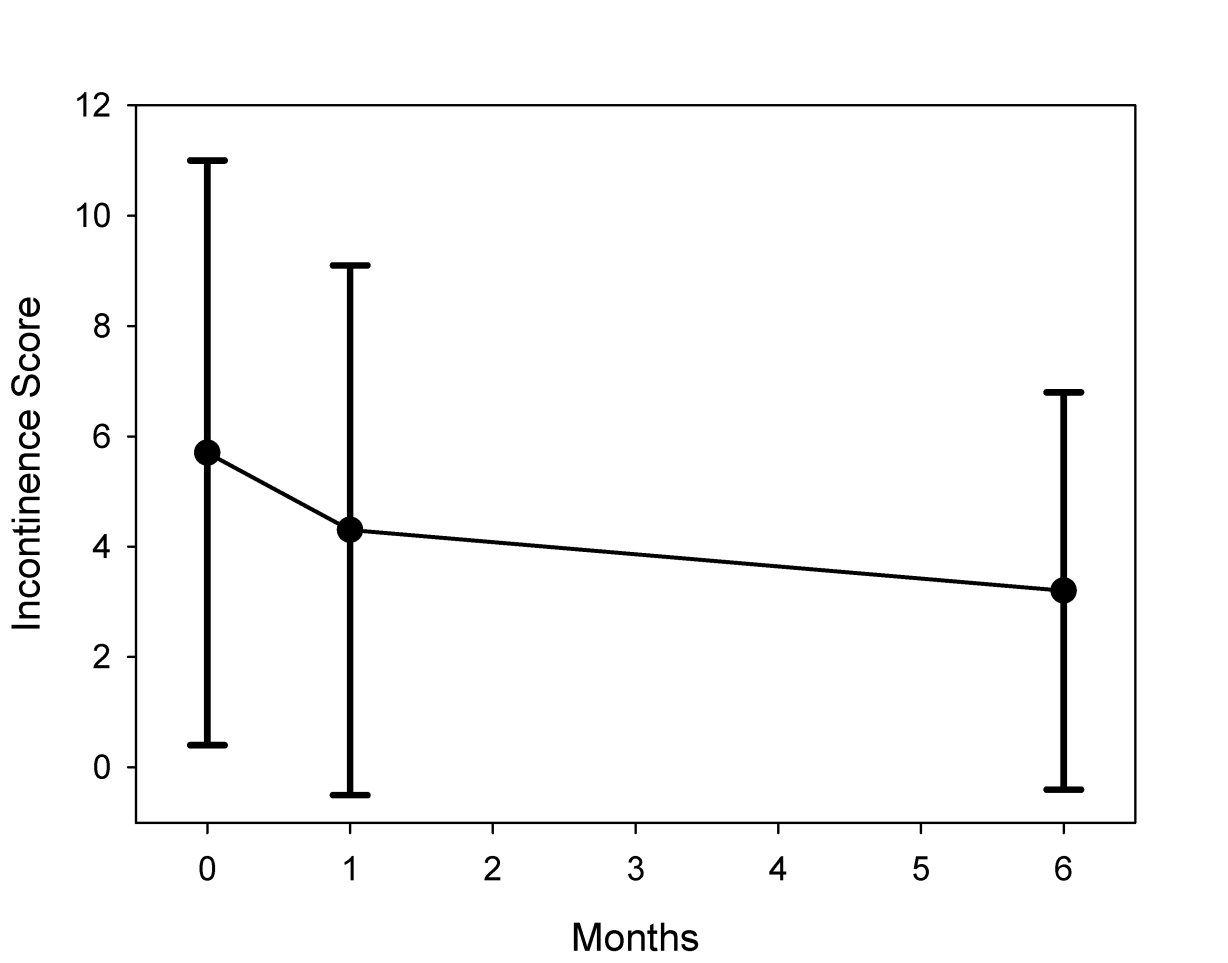
Cleveland Clinic Incontinence Score preoperative and at 1 month and 6 month follow-up

The Cleveland Clinic Incontinence Score performed in every patient improved from 5.7 (±5.2) to 4.3 (±4.8) 1 month postoperatively and 3.2 (±3.7), statistically significant to preoperative value (p<0.01) (Figure 4 (Fig. 4)).

Differentiation between the two subgroups of ODS and hemorrhoids shows the specimen weight was higher in the ODS group and that the stapling caused relief of the ODS symptoms in this group. Other variables did not show significant differences in both groups (Table 3 (Tab. 3)). 

## Discussion

Over the years, more than 100 different procedures have been described for surgical treatment of hemorrhoidal prolapse and functional disorders but the optimal surgical approach has yet not been determined [7]. In this context, stapled transanal procedures are one treatment option for low rectal prolapse and or functional disorders such as obstructed defecation. The purpose of this study was to evaluate the safety and efficacy of a new 36 mm stapling device allowing a full thickness rectal resection of the lower rectum. The multicenter setting was chosen in order to collect an adequate dataset in relative short time. The major endpoints of the study were initial perioperative variables such as technical problems with the stapling device itself and complication rate. Predominant endpoints of the follow-up analyses were the effect on the functional outcome represented by the changing of the defecation disorder und continence. In this respect the focus was on patients with the foremost symptom of obstructed defecation.

In order to evaluate the feasibility and early results a prospective multicenter study was brought up. Although, a multicenter setting always has the problem of potential inconsistencies, this setting gives the option to collect data in a relative short time. In addition, the majority of participant institutions already participated in the latter German STARR Registry Study Group and surgeons were well aware about the indication for STARR and the procedure and its pitfalls [5]. The evaluated endpoints were focusing on the surgical technique itself and the early results with special respect on pain and functional results. One of the major aspects was to evaluate whether the initial leading problem, prolapse or the functional disorder of rectocele and intussusceptions, improved. Although, it is a potential limitation of this study, that both hemorrhoidal prolapse and ODS were including criterias, this aspect also provides evidence on the quality of data collection. As expected, hemorrhoids were most often seen in men and ODS in women. In addition, as anticipated male hemorrhoidal prolapses were smaller and less tissue was removed compared to female patients. The effect of the stapling on functional disorders was much more pronounced in the ODS group.

The intraoperative problems were dominated by problems with introducing the stapler. This problem is relatively new and was not reported so often in other publications [5], [8]. Obviously, the problem of introducing the anvil of the stapler is mostly caused by the large diameter of 36 mm. As major perioperative difficulty partial dehiscence was reported in 4 cases. These were detected intraoperatively and closed transanally by full thickness rectal wall suturing. This result is in good agreement to other publications that found incomplete stapling only in a minority of cases [5]. Others found no dehiscence during surgery using the PPH01 stapler [9], [10]. An incomplete stapleline is most often caused by cutting through the rectal wall. If there is too much tissue between stapler and anvil, the rectal wall might be cut without adequate stapling [11]. 

One patient developed a postoperative partial insufficiency in the stapleline, which could be sealed by endoluminal vacuum therapy. Stapling insufficiency may, therefore, occur as in the classical surgical procedures, with stapled hemorrhoidopexy and STARR operation and other low rectal anastomosis [5], [12], [13]. 

Postoperative hemorrhage as the major cause for revision occurred in 6.3% of the observed population. This corresponds to the data from the literature with a postoperative hemorrhage rate of 3–12% [3], [10], [14]. Fortunately, no life-threatening bleedings occurred, as reported by others [15], [16], [17]. 

The purpose of prolapse removal was achieved in 95%. The symptoms of obstructed defecation syndromes also improved significantly. In agreement to other publications, the improvement remained in the early postoperative time [12]. Fecal incontinence, which seems quite possible, with respectively relevant anal dilatation by a 36 mm stapler, was not observed. 

Fecal urgency remains a problem in low rectal stapling procedures [18], [19]. The results of this prospective study coincide with the data of the Italian group of Naldini et al. [8]. Merely the urgency was much less frequently observed in the study by Naldini et al. than in this study. 

## Conclusion

The result of this prospective observational study shows the feasibility of transanal stapling using a new 36 mm stapling device (TST36). Windowed chamber and large specimen housing are new features of this stapler device that made resections up to 30 g rectal tissue possible. Nevertheless, the relatively large diameter of 36 mm caused technical problems in the introduction of the device. This may be an indication, that here it also reaches the limit of technical feasibility. Concerning the clinical results, a significant improvement of Altomare ODS score and Cleveland Clinic Incontinence Score was observed in short term follow-up. Although, the study demonstrated also a notable complication rate during surgery and the postoperative period, the number of reinterventions in anesthesia was small and serious and potentially life-threatening complications were an exception. Thus, the TST36 can be considered as a safe device and an effective tool for low rectal stapling for anorectal prolapse or obstructed defecation.

## Notes

### Competing interests

This study was supported by the manufacturer of the stapling device, Touchstone International Medical Science, Suzhou, China: the participating institutes were granted with € 200.- per patient.

The corresponding author received grant supply and serves as a consultant to Ethicon, Johnson & Johnson Medical GmbH, served as a consultant to Touchstone International Medical Science.

## Figures and Tables

**Table 1:**
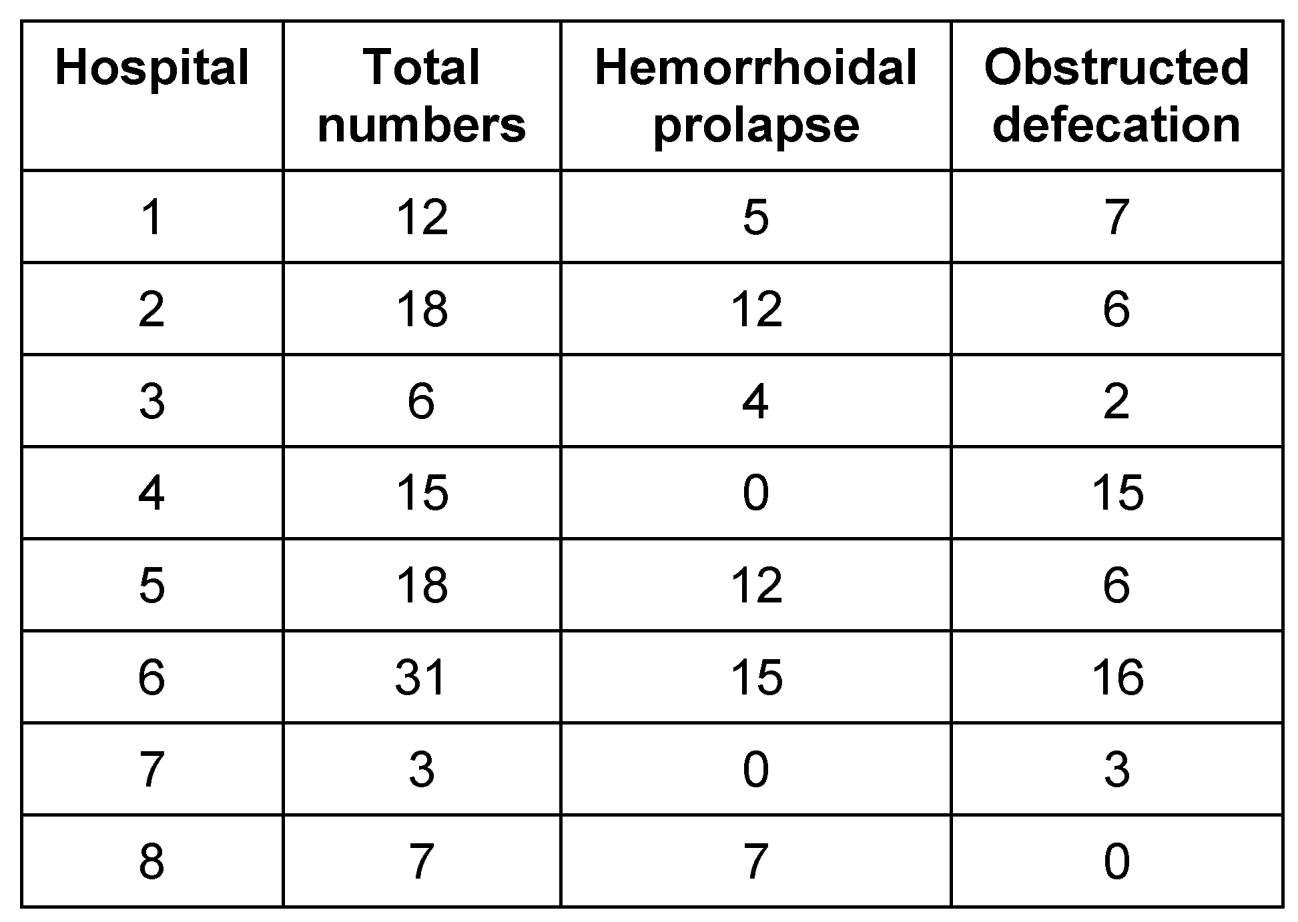
Number of patients, recruited in different institutes and indication for surgery, chi-square test (p=0.66)

**Table 2:**
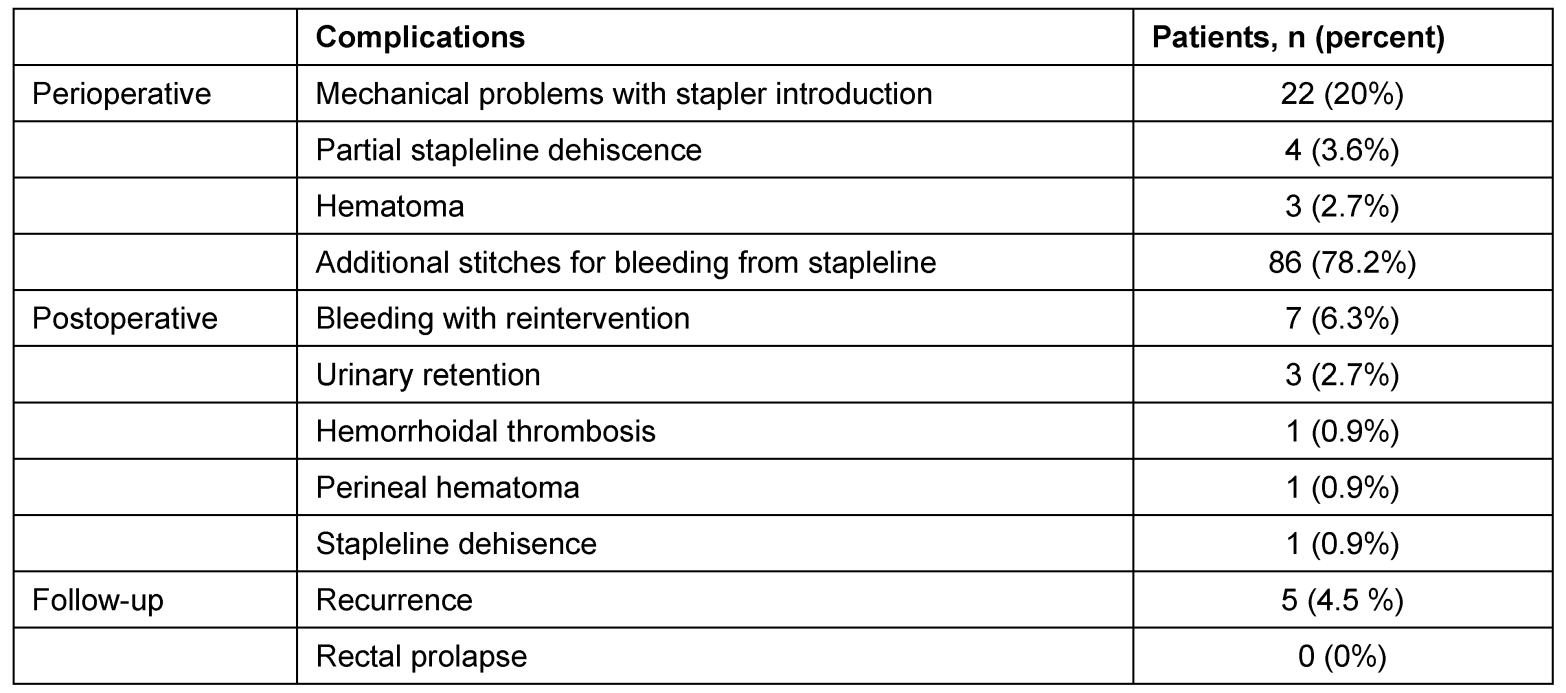
Complications

**Table 3:**
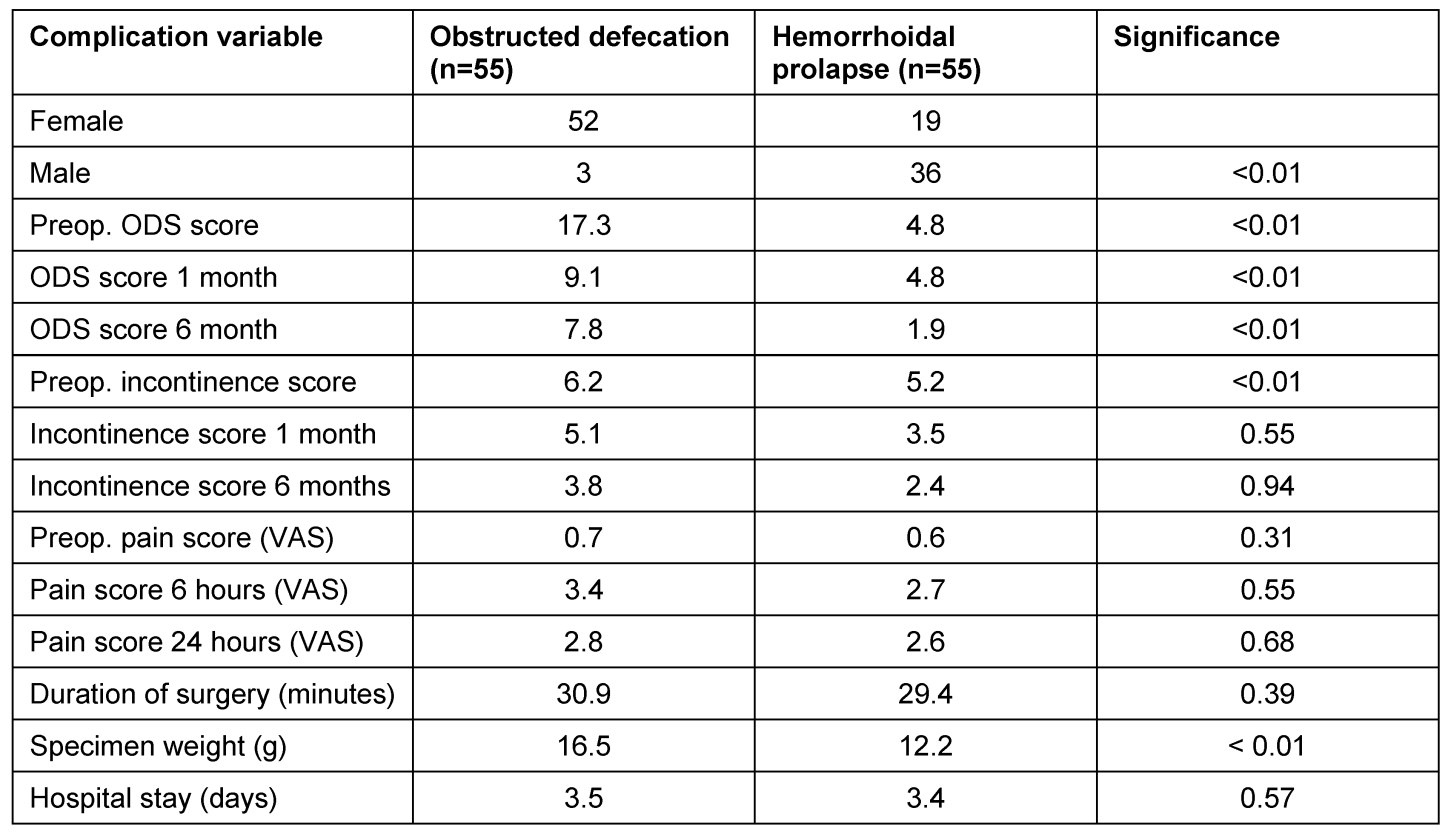
Patient characteristics in the groups with obstructed defecation and hemorrhoids
